# DiCE: differential centrality-ensemble analysis based on gene expression profiles and protein–protein interaction network

**DOI:** 10.1093/nar/gkaf609

**Published:** 2025-07-08

**Authors:** Elnaz Pashaei, Sheng Liu, Kailing Li, Yong Zang, Lei Yang, Tim Lautenschlaeger, Jun Huang, Xin Lu, Jun Wan

**Affiliations:** Department of Medical and Molecular Genetics, Indiana University School of Medicine, Indianapolis, IN 46202, United States; Department of Medical and Molecular Genetics, Indiana University School of Medicine, Indianapolis, IN 46202, United States; Department of BioHealth Informatics, Luddy School of Informatics and Computing, Indiana University at Indianapolis, Indianapolis, IN 46202, United States; Department of Biostatistics and Health Data Science, Indiana University School of Medicine, Indianapolis, IN 46202, United States; Center for Computational Biology and Bioinformatics, Indiana University School of Medicine, Indianapolis, IN 46202, United States; Indiana University Simon Comprehensive Cancer Center, Indiana University School of Medicine, Indianapolis, IN 46202, United States; Center for Computational Biology and Bioinformatics, Indiana University School of Medicine, Indianapolis, IN 46202, United States; Department of Pediatrics, Herman B Wells Center for Pediatric Research, Indiana University School of Medicine, Indianapolis, IN 46202, United States; Indiana University Simon Comprehensive Cancer Center, Indiana University School of Medicine, Indianapolis, IN 46202, United States; Department of Radiation Oncology, Indiana University School of Medicine, Indiana University School of Medicine, Indianapolis, IN 46202, United States; Pritzker School of Molecular Engineering, University of Chicago, Chicago, IL 60637, United States; Indiana University Simon Comprehensive Cancer Center, Indiana University School of Medicine, Indianapolis, IN 46202, United States; Department of Biological Sciences, Boler-Parseghian Center for Rare and Neglected Diseases, Harper Cancer Research Institute, University of Notre Dame, Notre Dame, IN 46556, United States; Department of Medical and Molecular Genetics, Indiana University School of Medicine, Indianapolis, IN 46202, United States; Department of BioHealth Informatics, Luddy School of Informatics and Computing, Indiana University at Indianapolis, Indianapolis, IN 46202, United States; Center for Computational Biology and Bioinformatics, Indiana University School of Medicine, Indianapolis, IN 46202, United States; Indiana University Simon Comprehensive Cancer Center, Indiana University School of Medicine, Indianapolis, IN 46202, United States

## Abstract

Uncovering key genes that drive diseases and cancers is crucial for advancing understanding and developing targeted therapies. Traditional differential expression analysis often relies on arbitrary cutoffs, missing critical genes with subtle expression changes. Some methods incorporate protein–protein interactions (PPIs) but depend on prior disease knowledge. To address these challenges, we developed DiCE (Differential Centrality-Ensemble analysis), a novel approach that combines differential expression with network centrality analysis, independent of prior disease annotations. DiCE identifies candidate genes, refines them with an information gain filter, and reconstructs a condition-specific weighted PPI network. Using centrality measures, DiCE ranks genes based on expression shifts and network influence. Validated on prostate cancer datasets, DiCE identified genes overrepresented in key pathways and cancer fitness genes, significantly correlating with disease-free survival (DFS), despite DFS not being used in selection. DiCE offers a comprehensive, unbiased approach to identifying disease-associated genes, advancing biomarker discovery and therapeutic development.

## Graphical Abstract

**Graphical Abstract:**
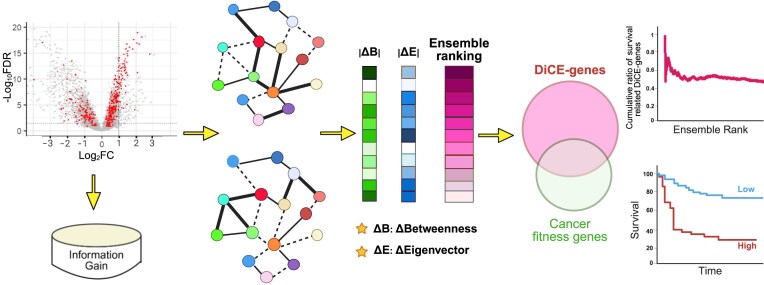

## Introduction

Differential expression analysis (DEA) is a pivotal step in pinpointing differentially expressed genes (DEGs) with expression levels systematically changed between two distinct conditions. Recently, machine learning techniques have been increasingly applied to analyze genomic data for identifying DEGs. However, these methods predominantly rely on statistical models to analyze gene expression data and identify DEGs at the individual gene level. Especially, DEGs are often determined by arbitrarily chosen cutoffs, e.g. *P*-value or false discovery rate-adjusted *P*-value (*q*-value) below a specific value, typically .05, and/or expression fold change (FC) exceeding a predefined threshold (commonly |log_2_FC| > 1). While effective, such strict cutoffs risk overlooking biologically significant genes that might not exhibit substantial expression changes but play crucial roles in biological functions and pathways related to diseases or phenotypes. Alternative approaches, such as weighted gene co-expression network analysis [1], model-based gene clustering (MBCdeg) [2], and Bayesian model-based multi-tissue clustering algorithm (revamp) [3], categorize genes based on the similarity of expression patterns. However, focusing on individual gene levels without accounting for protein–protein interactions (PPIs) may fail to identify gene signatures with pivotal roles within the comprehensive gene regulatory network. Some methodologies such as PhenoGeneRanker [4], user-guided knowledge-driven integrative network-based method [5], and prioritization with a warped network [6] employ disease-specific genes to navigate through the biological networks, e.g. PPIs, in order to prioritize genes associated with specific diseases. These approaches typically depend on a predefined set of disease-specific genes, often called “seed genes,” known to be associated with the disease under investigation [7]. If the information about seed genes is either absent or biased, the results may be inaccurate or incomplete.

To address these constraints and enhance the precision of gene prioritization to better identify key genes that are more contextually relevant with biological significance, we developed an innovative approach called differential centrality-ensemble (DiCE). DiCE harnesses multiple components, beginning with DEA to construct a candidate gene pool, followed by feature selection using information gain (IG) to retain the most informative genes. Next, it incorporates topological assessment of sample-specific weighted PPI networks to capture condition-specific interactions. Centrality measures, such as betweenness and eigenvector centrality, are employed to assess genes’ network influences. Betweenness centrality identifies genes acting as bridges between functional modules, while eigenvector centrality highlights genes connected to other highly influential nodes. Changes in these metrics between conditions, e.g. tumor versus normal or treated versus untreated, can reveal functional rewiring of the network. An increase in centrality might suggest that a gene has gained importance or interactions, whereas a decrease could indicate diminished influence or regulatory disruption. These network-based changes can uncover key genes involved in cancer/disease progression or therapeutic response that might be overlooked by DEA alone. To synthesize these insights, DiCE applies an ensemble ranking strategy to combine multiple prioritization criteria, resulting in a refined set of DiCE genes. This comprehensive framework enhances the robustness and interpretability of the gene selection process by integrating statistical significance with biological relevance.

The efficiency of DiCE was assessed with two prostate cancer (PCa) datasets, including PCa tumor versus normal samples and PCa metastatic tumors versus primary tumors. DiCE effectively identifies and prioritizes not only disease-candidate genes but also survival-correlated and fitness genes. These findings underscore DiCE’s potential to identify gene signatures linked to cancer mechanisms and prognostic significance, thus offering valuable insights into clinical applications in cancer management. Notably, DiCE operates independently of known disease-related genes, making it applicable even in contexts with limited prior knowledge.

## Materials and methods

### Workflow of DiCE

**Figure 1.**
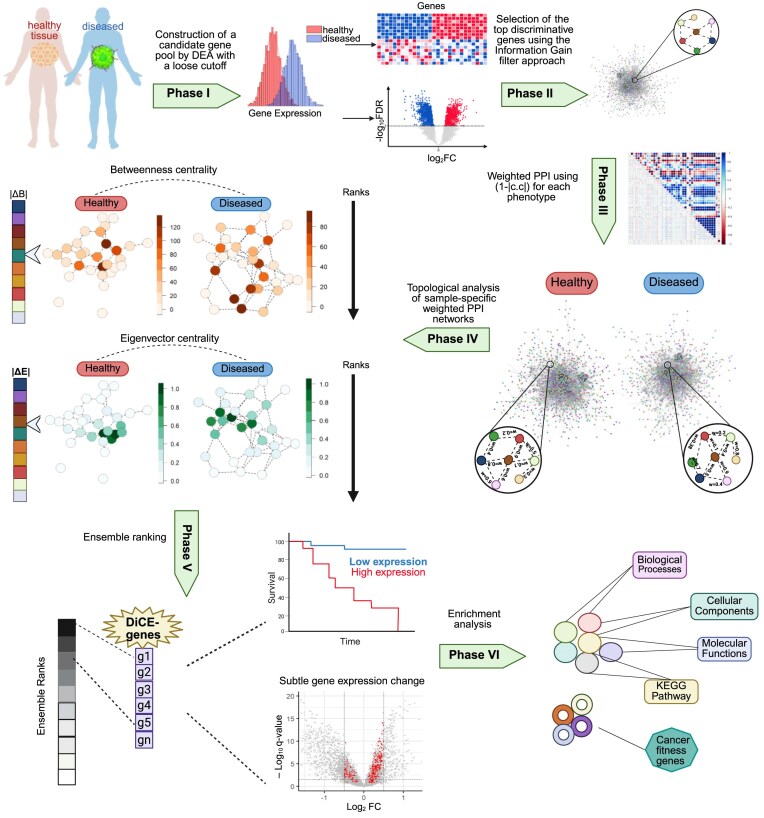
DiCE workflow.

The DiCE algorithm mainly consists of six phases (Fig. 1). Phase I: construction of a candidate gene pool by DEA with relaxed cutoffs, such as *P* < .05 with or without an FC threshold. Phase II: selection of the top discriminative genes from the candidate pool obtained in Phase I using the IG filter approach. Phase III: modification of weighted PPI network by calculating Pearson correlation coefficients (c.c.) of gene pairs in terms of gene expression for each specific phenotype or sample type. The value of (1 − |c.c.|) is assigned as the normalized distance between paired genes in accordance with the PPI network. Phase IV: topological analysis of sample-specific weighted PPI networks from the perspectives of betweenness and eigenvector centrality, followed by differential analysis of each centrality measure for individual genes. Phase V: ensemble ranking to produce a reliable and robust rating by integrating the ranks established in the previous stage. Phase VI: enrichment analysis of biological pathways and functions for identified DiCE genes.

### Phase I: construction of gene candidate pool by DEA

DEA serves as the first step in the DiCE, generating a large candidate gene pool that forms the foundation for subsequent phases. In this study, DEA is performed using lmFit and eBayes functions in limma-voom [8] for the microarray dataset (GSE21032) [9] or edgeR package [10] for the RNA-seq data of prostate adenocarcinoma (PRAD) cohort [11] in the TCGA (The Cancer Genome Atlas). To capture genes with subtle expression changes, we applied relatively loose cutoffs that allow modest FCs and less statistical significance with higher *P*-values. The subsequent steps from Phase II to Phase VI, including IG filtering, reconstruction of the weighted PPI network, and differential centrality analysis, serve as progressive layers of refinement to prioritize genes with significant topological and functional changes. These multistep strategies operate as orthogonal complements to traditional DEA, helping to uncover genes that may show limited expression changes yet play pivotal roles in network dynamics and cancer or disease progression, while also minimizing the retention of spurious associations.

### Phase II: selection of most discriminative genes using IG

The IG filter is a widely used statistical measure for gene selection [12]. It quantifies the amount of information a gene contributes to differentiating between conditions. Genes with higher IG values tend to be more informative and effective in distinguishing between groups.

Consider a dataset with $N$ instances distributed across $k$ classes. The ratio of samples in each class is denoted as ${{{S}_i}}/{N}$, where ${S}_i$ ($i\ = \ 1,\ 2,...,$  $k$) represents the number of samples in the ${i}{{\rm th}}$ class out of *N* total samples. The initial entropy *D* of the dataset, prior to any split, is calculated as follows:


(1) \begin{eqnarray*}
D = - \mathop \sum \limits_{i = 1}^k \ \frac{{{S}_i}}{N} \times {{\rm log}}_2\frac{{{S}_i}}{N}.
\end{eqnarray*}

Given a gene *g* with different expression levels $V = \{ {{v}_1,\ {v}_2,\ldots ,{v}_m} \},$ where *v_i_* denotes the distinct expression levels and *m* corresponds to the number of *v_i_*, the weighted entropy of gene *g* is calculated as follows:


(2) \begin{eqnarray*}
{\rm weighted}\ {\rm entropy}\left( g \right) = - \mathop \sum \limits_{v = 1}^m \frac{{\left| {{N}_v} \right|}}{N} \times {\rm entropy}\left( {{N}_v} \right),
\end{eqnarray*}

where ${N}_v$ represents the partitioned subset of *N* samples with the size of ${\mathrm{\ }}| {{N}_v} |,$ based on expression values of gene *g*. Finally, the IG value of gene *g* can be calculated as follows:


(3) \begin{eqnarray*}
{\rm IG}\left( g \right) = D - {\rm weighted}\ {\rm entropy}\left( g \right).
\end{eqnarray*}

In fact, IG quantifies the reduction in entropy by measuring how well the expression levels of individual genes can partition samples into distinct classes. A higher IG indicates a greater ability to distinguish between classes, thereby reducing uncertainty (entropy) in classification. The candidate gene pool refined by the IG filter keeps the most informative genes, characterized by their high discriminative powers. Specifically, genes are selected for the next analysis step if their IG values exceed the average IG value of all genes in the pool. This enhances interpretability, boosts classification performance, and optimizes computational efficiency, ultimately leading to a more effective analysis.

### Phase III: modification of PPI network with gene expression correlations

The PPI networks provide a comprehensive view of the connections among genes. Genes are depicted as vertices linked by undirected edges that represent their interactions. Despite variations in biological systems being studied, the STRINGdb package [13] in R (v.11) for human (species = 9606) with a confidence score threshold of 0.4 serves as the resource of PPIs.

To identify biologically relevant crucial genes among the top discriminative genes selected in Phase II, a systematic approach was employed according to their connectivity and interaction patterns. Given the Pearson correlation coefficients (c.c.) of gene expression for specific conditions or sample types between pairs of genes in Phase II, the value of $( {1 - | {{\rm c.c.}} |} )$ was used as a distance-like metric to characterize the degree of dissimilarity or lack of connectivity between nodes/genes to discover the shortest paths between network nodes. By generating condition/sample type-specific PPI networks, we can effectively capture the differentiation between various conditions or samples from the perspective of the strength of gene relationships within the networks.

### Phase IV: topological analysis of sample-specific weighted PPI networks using betweenness and eigenvector centrality

Topological analysis examines various network metrics and properties to gain insights into its key structure and characteristics. For example, centrality metrics can pinpoint proteins that hold the most central or influential positions in the network and highlight proteins acting as bridges or are crucial for the flow of information.

In this study, two distinct centrality measures, betweenness and eigenvector, were employed to assess node importance and significance within the condition/sample-specific weighted PPI networks. Betweenness centrality gauges the proportion of the number of shortest paths that traverse each node, providing an estimate of the frequency of the node lying on these paths between other nodes. The distances from or to the network’s vertices are used to calculate the shortest path. Higher betweenness centrality of one node indicates its greater impact on information flow within the network. It effectively identifies bottlenecks and evaluates the global importance of nodes. The robustness of betweenness centrality in the identification of target genes has been attested by many studies. Eigenvector centrality extends from the degree centrality by considering a node’s importance based on its connections to other important nodes. This metric accounts for both the number of connections and the importance and significance of nodes within the network by taking into consideration the degrees of neighboring nodes.

A node with numerous connections might have a relatively low eigenvector centrality if its connections link to nodes with low degrees. Similarly, a node could possess a high betweenness but a low eigenvector centrality, indicating that it is distant from the network’s influential nodes and bridges disparate parts within the network. Combining both eigenvector and betweenness centralities in network analysis provides complementary insights into node importance and influence and enhances our ability to identify and interpret key features within the network, thereby facilitating a deeper understanding of its intricate relationships, uncovering hidden patterns from gene regulatory network perspective, and elucidating the underlying mechanisms driving its behavior. By leveraging eigenvector and betweenness centralities, we are better equipped to discern critical genes, detect community structures, and grasp the flow of information or influence across the network.

### Phase V: DiCE genes identified by gene prioritization using ensemble ranking based on absolute differences of each centrality measure

Considering the betweenness and eigenvector centralities of each gene within weighted PPI networks under distinct conditions or samples, such as normal and tumor samples or groups before and after treatment, we computed the absolute differences in each centrality measure between these various conditions/samples. Evaluating the absolute disparities in two centrality metrics enables us to spotlight genes that experienced notable shifts in their centralities, potentially indicating significantly altered roles or functional relevance within specific conditions or among distinct sample types. While both betweenness and eigenvector centralities were employed to evaluate condition/sample-specific weighted PPI networks, the absolute values of differences in either one of them yielded distinct rankings. This underscores the necessity of establishing an overall aggregated ranking of alternatives. Subsequently, the genes were ranked in descending order based on their absolute differences for each centrality measure. The genes with the greatest absolute differences were most likely important with pivotal roles associated with distinct groups/conditions, signifying their prominences and impacts within the context of condition or sample-specific weighted PPI networks, in addition to differential gene expression levels. Ensemble ranking, a technique in data analysis and decision-making, merges multiple individual rankings into a unified, aggregated ranking that enhances the overall accuracy, resilience, and reliability of the final gene ranking by harnessing the combined strengths of various ranking strategies. We adopted an ensemble ranking strategy inspired by the product of rank method [14]. Each gene was ranked according to its centrality changes across multiple network centrality measures. To integrate these rankings, we normalized each to a [0, 1] scale, where 1 represents the highest importance and 0 the lowest, to account for differences in scale among metrics. The final score for each gene was then computed as the product of its normalized ranks across all selected metrics. This method emphasizes genes that consistently rank high across multiple criteria, while preventing any single metric from dominating the ensemble ranking.

The DiCE genes were finally identified after excluding those with centrality measures lower than the average value in both phenotypes or conditions. This cutoff mirrors the one used in Phase II for IG filtering. By relying on the mean value as a threshold, we implemented a straightforward, interpretable, and data-driven method to prioritize more informative or influential genes at each stage. Using the mean, rather than a fixed value or percentile, ensures adaptability across datasets of different sizes and characteristics. Although more complex thresholds are possible, e.g. based on distribution extremes, the mean-based criterion proved effective in eliminating low-information or peripheral genes without being overly restrictive.

### Phase VI: biological function and pathway enrichment analysis

Enrichment analysis of biological functions and pathways was conducted to determine and interpret the comprehensive functional roles of DiCE genes, providing more insights into the underlying molecular mechanisms responsible for the differences observed among distinct conditions/samples in the study. To identify Gene Ontology (GO) terms and KEGG (Kyoto Encyclopedia of Genes and Genomes) pathways significantly enriched in the identified DiCE genes, the interactive tool DAVID (Database for Annotation Visualization and Integrated Discovery, https://david.ncifcrf.gov/) was employed. Only GO terms and KEGG pathways with false discovery rate (FDR) less than 0.05 were considered significantly enriched.

### Survival analysis

The survival analysis was performed using a Cox proportional hazards model by stratifying all genes into high- or low-expression group based on the median expression levels. Our study utilized disease-free survival (DFS) information of 500 PRAD patients in the “Prostate Adenocarcinoma (TCGA, Firehorse Legacy)” dataset [15] with corresponding clinicopathologic information available on cBioPortal (https://www.cbioportal.org/) [15, 16]. Gene with a *P*-value <.05 in the survival analysis was considered as survival associated.

## Results

### DiCE analysis on the TCGA PCa dataset

**Figure 2.**
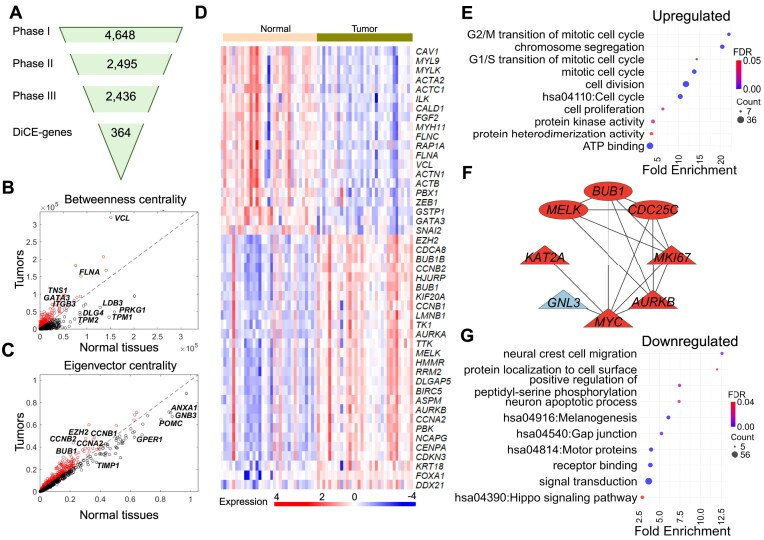
Application of DiCE to PCa tumor versus normal tissues. (**A**) Number of genes retained in each phase of the proposed approach. Betweenness (**B**) and eigenvector (**C**) centrality for each gene in normal tissues (*x*-axis) and PCa tumors (*y*-axis). (**D**) Expression levels of DiCE genes in normal tissues and tumors, which were identified as hub genes in previous studies. (**E**) Selected GO terms and KEGG pathways significantly enriched in upregulated DiCE genes. (**F**) PPI network of upregulated DiCE genes involved in cell proliferation. Node shapes represent changes in betweenness between tumor and normal samples (triangle for an increase and oval for a decrease), while the red and blue colors indicate a positive and negative shift in eigenvector centrality, respectively. (**G**) Selected GO/KEGG pathways notably overrepresented in downregulated DiCE genes.

To perform the differential ensemble centrality analysis on the TCGA PCa dataset, we first obtained gene expression data from tumor and adjacent normal prostate samples using the TCGA platform (https://gdac.broadinstitute.org/) [11]. The dataset included 33 patients, each contributing tumor and paired normal tissue samples. Preprocessing steps involved removing noncoding genes (long non-coding RNA and pseudogenes), resulting in an expression profile of 16 322 genes across tumor and normal samples. Figure 2A summarizes the numbers of selected genes at each phase by the DiCE analysis applied to the TCGA PCa dataset. More details are provided below.

Phase I constructed the pool of candidate genes. DEA was performed using the edgeR [10] for paired comparison between PCa tumors and adjacent normal samples to select candidate genes based on loose cutoffs: FDR < 0.05 and |log_2_FC| > 0.5, resulting in 4648 candidate genes in the pool (Fig. 2A). *Z*-score normalization was then applied to the expression data of selected candidate genes for downstream analysis.

In Phase II, the IG filter was applied to all candidate genes to identify the most discriminative genes from the candidate pool. Each gene was assigned a weight based on its entropy. A total of 2495 genes with weights higher than the mean weight were identified as the most discriminative genes for further analysis (Fig. 2A).

In Phase III, the PPI network of the most discriminative genes based on the STRING database [13] was adjusted based on gene expression correlations across different conditions or samples. Correlations were treated as a distance-like metric, calculated as $( {1 - | {{\rm c.c.}} |} )$, in the PPI network. After excluding 59 genes that lacked connections, the resulting sample-specific weighted PPI networks included 2436 genes (Fig. 2A) and 19 472 interactions.

Phase IV included a topological analysis of the sample-specific weighted PPI networks using two centrality measures: betweenness and eigenvector. These parameters were calculated for each gene (node) within the network for both tumor and normal samples.

An increase in the betweenness centrality of a node/gene in tumor samples indicates that the gene lies on shorter paths connecting other genes, suggesting its role as a bridge in tumor-related PPIs (Fig. 2B), for instance, *VCL*, *FLNA*, *TNS1*, *GATA3*, and *ITGB3*, which may act as key connectors in tumor samples. Conversely, decreased betweenness centralities observed for genes like *LDB3*, *PRKG1*, *TPM1*, *TPM2*, and *DLG4* suggest reduced involvement in shortest paths or a diminished role in connecting to other genes (Fig. 2B).

Eigenvector centrality measures the relative influence of a node/gene within the network (Fig. 2C). An increase in eigenvector centrality indicates connections to highly influential genes, enhancing the gene’s significance in the network as *EZH2*, *CCNB1*, *CCBB2*, *CCNA2*, and *BUB1* in PCa tumors. On the other hand, a decrease in eigenvector centrality indicates that a gene is connected to less influential or weakening genes, possibly resulting in lower relative influence scores or reduced significance in the network, such as *ANXA1*, *GNB3*, *POMC*, *GPER1*, and *TIMP1* in tumor samples (Fig. 2C).

In Phase V, ensemble ranking was used to prioritize genes based on the absolute differences of each centrality measure from Phase IV. Genes were excluded if their centrality measures in both sample groups/types/conditions were below the average value for the corresponding group. This process identified 364 DiCE genes, comprising 138 upregulated and 226 downregulated genes in PCa tumors. These DiCE genes demonstrated notable alterations of these two centrality scores, along with gene expression changes between tumor and normal samples, highlighting their crucial role in shaping the network structure and influencing other genes in response to condition changes. Among them, 47 DiCE genes were recognized as hub genes (Fig. 2D) in multiple PCa studies [17–26].

We performed functional enrichment analysis on up- and downregulated DiCE genes in terms of GO and KEGG pathways. The 138 upregulated genes were enriched in processes such as cell proliferation, cell cycle, cell division, chromosome segregation, protein kinase activity, ATP binding, protein heterodimerization activity, and multiple mitotic-related biological processes, e.g. mitotic cell cycle (Fig. 2E). Eight of these upregulated DiCE genes, *KAT2A* (lysine acetyltransferase 2A), *MELK*, *MYC*, *MKI67*, *CDC25C*, *BUB1*, *AURKB*, and *GNL3*, are associated with cell proliferation (Fig. 2F). Among them, five genes showed higher betweenness (triangle) in PCa tumor and almost all of them exhibited increased eigenvector centrality (red color). These findings underscore their roles as central hubs orchestrating broader transcriptional, signaling, or regulatory changes.

In contrast, 226 downregulated genes were associated with multiple KEGG pathways such as melanogenesis, gap junction, Hippo signaling pathways, and biological processes, e.g. signal transduction, receptor binding, neural crest cell migration, protein location to cell surface, neuron apoptotic process, etc. (Fig. 2G). The Hippo pathway is crucial in PCa and modulates androgen receptor (AR) signaling via *YAP*, impacting AR + PCa growth [27]. Additionally, 12 out of 15 downregulated motor proteins exhibited reduced betweenness centrality in PCa tumors, including *TPM1* and *TMP2* (Fig. 2B), both recognized as tumor suppressors, further indicating a disruption of tumor-suppressor circuits during PCa progression and highlighting potential therapeutic targets.

To assess the robustness of our PPI-based results, we repeated the DiCE analysis using a more stringent STRING confidence score threshold of 0.7, compared to the original medium threshold of 0.4. This stricter cutoff yielded 66 DiCE genes, 55 of which were identified in the original analysis (Supplementary Fig. S1A). These 55 genes tended to rank higher than those not retained under the higher threshold (Supplementary Fig. S1B), indicating stronger interaction support. While the number of interactions and identified genes decreased under the stricter cutoff, the overall centrality aberration patterns remained consistent, underscoring the reliability and reproducibility of the DiCE across different stringency levels of PPI network.

### DiCE analysis on metastatic PCa data

**Figure 3.**
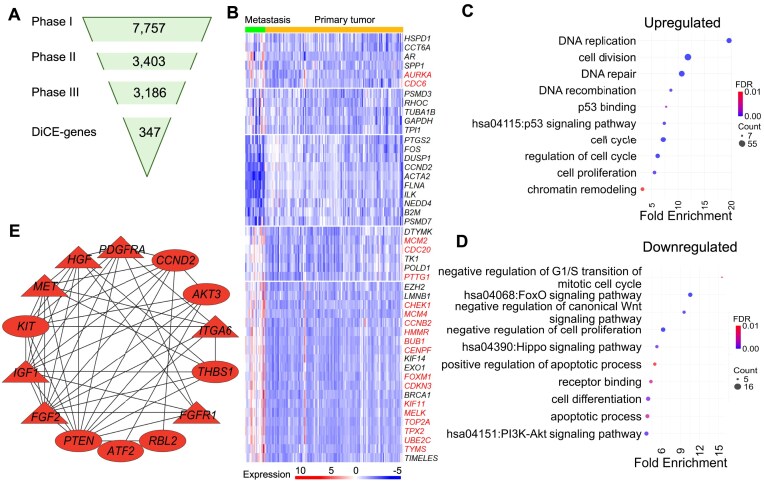
Application of DiCE to PCa metastasis versus primary tumors. (**A**) Number of genes in each phase of DiCE. (**B**) Expression of 47 selected DiCE genes associated with PCa metastasis previously identified through a meta-analysis of five studies. Nineteen hub genes are marked in red. Enriched GO terms and KEGG pathways in upregulated (**C**) and downregulated (**D**) DiCE genes. (**E**) PPI network for downregulated DiCE genes in the PI3K-Akt signaling pathway. Node shapes and colors follow the same scheme as in Fig. 2F.

We applied DiCE to the GSE21032 dataset [9], comprising 131 primary and 19 metastatic PCa tumors. DEA by limma identified 7757 candidate genes with a loose cutoff of *P*-value <.05 (Fig. 3A). A subset of 3403 genes was identified as the most discriminative in Phase II using the IG filter, followed by reconstruction of the PPI network (Phase III) keeping 3186 genes with modified node distances using (1 − |c.c.|) based on gene expression profiles in metastatic PCa and primary tumors, respectively. Topological differential analysis in Phase IV further narrowed the list to 347 DiCE genes in Phase V (Fig. 3A), encompassing 47 genes identified through a meta-analysis of five studies [28], including 19 hub genes (Fig. 3B).

Two hundred thirty-eight upregulated DiCE genes in PCa metastasis were significantly enriched in cell cycle, cell division, cell proliferation, DNA repair/replication/recombination, and chromatin remodeling (Fig. 3C). Strikingly, several genes involved in p53 binding and p53 signaling pathway, including *GTSE1*, *RRM2*, *CCNB1*, and *CHEK1*, were upregulated in metastatic PCa. *GTSE1*, which is highly expressed in various cancers, including PCa, and linked to poor prognoses [29], promotes PCa cell proliferation by activating the SP1/FOXM1 signaling pathway [30]. Similarly, *RRM2* overexpression was associated with aggressive PCa and unfavorable outcomes [31]. Despite their elevated expression, both *GTSE1* and *RRM2* exhibited reduced betweenness centrality in PCa metastasis compared to primary tumors. Moreover, the combined overexpression of *RRM2* and *CCNB1* has been implicated in driving PCa progression via the cell cycle and the p53 signaling pathway [31]. While *CCNB1* and *CHEK1* showed decreased eigenvector centrality in metastasis, *CHEK1* plays a key role in the DNA damage response [32] and contributes to tumor progression and therapy resistance in PCa, particularly in metastatic castration-resistant prostate cancer (CRPC) [33].

The 109 downregulated DiCE genes are involved in a wide range of biological processes (Fig. 3D), e.g. apoptotic process, cell differentiation, receptor binding, negative regulation of cell proliferation, and negative regulation of canonical Wnt signaling pathway, besides Hippo, FoxO, and PI3K-Akt signaling pathways. Fourteen of these DiCE genes, *PDGFRA*, *ATF2*, *HGF*, *PTEN*, *IGF1*, *FGF2*, *THBS1*, *RBL2*, *CCND2*, *KIT*, *AKT3*, *ITGA6*, *MET*, and *FGFR1*, are linked to the PI3K-Akt pathway, a key oncogenic driver of migration, proliferation, and drug resistance, frequently dysregulated in metastatic PCa [34]. Among them, *PTEN* is a well-established tumor suppressor in PCa, acting as a major inhibitor of growth signaling and interacting with the MAPK pathway through PPIs [35]. Oncogenes, such as *FGF2*, *FGFR1*, *HGF*, *IGF1*, *MET*, and *PDGFRA*, showed higher betweenness (triangles in Fig. 3E) in metastatic PCa, despite their reduced gene expression. Additionally, all 14 DiCE genes displayed increased eigenvector centrality (red color in Fig. 3E), emphasizing their enhanced influence within the network during PCa metastasis.

### DiCE genes are more likely correlated with patient survival outcomes

**Figure 4.**
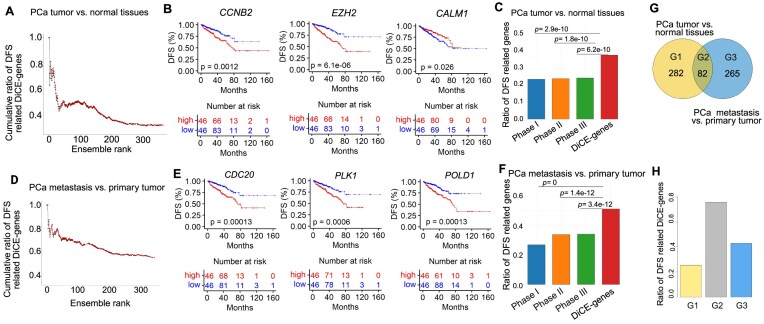
Association between key genes and patients’ survival outcomes. (**A**) Cumulative ratios of DiCE genes for PCa tumor versus normal tissues that were significantly associated with DFS along with their ensemble rankings. (**B**) KM plots of top-ranked DiCE genes with significant survival outcomes. (**C**) Ratio of DFS-related genes in each phase of DiCE. (**D**) Cumulative proportions of DFS-related DiCE genes for PCa metastasis versus primary tumors against their rankings. (**E**) KM plots of selected top-ranked DiCE genes from the metastasis analysis. (**F**) Ratio of DSF-related genes in each DiCE phase. The statistical significance of the ratio differences in panels (C) and (F) was evaluated based on hypergeometric distribution. (**G**) Overlap of two sets of DiCE genes derived from PCa tumor versus normal tissues and metastasis versus primary tumors. (**H**) Ratio of DFS-related DiCE genes in each segment shown in panel (G).

Next, survival analysis was conducted on DiCE genes using the Cox proportional hazards model. Among 364 DiCE genes identified upon the comparison between PCa tumors and normal samples, 134 (36.8%) presented significant associations (*P* < .05) between their expression levels and patients’ DFS, even though DFS information was not included during DiCE gene selection. Cumulative ratios of DiCE genes associated with DFS outcomes, along with their final DiCE rankings, revealed that lower gene ensemble ranks led to a decline in the proportion of survival-correlated genes (Fig. 4A). These suggest that top-ranked DiCE genes tend to be more correlated with clinical outcomes, such as *CCNB2* (rank #1), *EZH2* (rank #2), and *CALM1* (rank #4) (Fig. 4B). Cyclin B2 (*CCNB2*), a key regulator of the cell cycle, has been reported to be upregulated in human cancers. Its increased expression is associated with poor survival outcomes, suggesting its potential as a novel prognostic maker. *EZH2* plays a multifaceted role in PCa progression. Many studies have demonstrated that the polycomb repressive complex 2 (PRC2)-dependent transcription repression by *EZH2* suppresses interferon γ-signaling and promotes PCa cell invasion, cancer stem cell features, and angiogenesis, among others. The aberrant expression of *CALM1* was detected in several cohorts of PCa patients.

For comparison, ratios of genes linked to patient outcomes at each phase of the DiCE were calculated (Fig. 4C). Only 22.4% of 4648 candidate genes after Phase I (DEA) showed survival significance, along with 22.9% and 23.3% of genes from Phase II and Phase III of DiCE, respectively. All of them were notably lower than the ratio of DiCE genes according to the hypergeometric test.

A similar trend was observed for the DiCE genes distinguishing metastasis and primary PCa. Fifty-one percent of 347 DiCE genes were significantly associated with DFS outcomes (Fig. 4D), including top ranked DiCE genes, such as *CDC20* (rank #1), *PLK1* (rank #3), and *POLD1* (rank #14) (Fig. 4E). *CDC20* and *PLK1* are important biomarkers and potential therapeutic targets for metastatic PCa. The diverse expression profiles of *POLD1* (DNA polymerase delta 1 catalytic subunit gene) in various tumor types indicate its potential significance across different cancers as reported in previous studies, even though its specific role in PCa remains less defined. However, in our study, *POLD1* explicitly exhibited a favorable rank and a highly significant survival *P*-value, indicating its potential as a prognostic marker or therapeutic target in PCa. Again, the survival significance ratio of DiCE genes in Phase V was considerably higher than in any prior phases (Fig. 4F).

Of the 347 DiCE genes distinguishing PCa metastatic from primary tumors, 82 were also identified among the 364 DiCE genes in the comparison between PCa tumors and normal samples (Fig. 4G). These overlapping genes exhibited consistent expression patterns, either up- or downregulated, in both comparisons (Supplementary Fig. S2). The significant overlap highlights the dual roles of genes such as *TOP2A*, *CCNB2*, *BUB1*, *TPX2*, and *EZH2* in both tumor initiation and tumor progression, reinforcing their potential as key biomarkers for PCa. This consistent identification across different comparisons strengthens their relevance for further experimental validation and therapeutic exploration. Importantly, 62 out of these 82 shared DiCE genes (75.6% of G2) were significantly associated with DFS (Fig. 4H), emphasizing their strong prognostic potential and roles in understanding PCa mechanisms. In comparison, lower proportions of DiCE genes linked to survival significance were observed in DiCE genes exclusively in PCa tumor versus normal (72 out of 282 in G1, 25.5%) or PCa metastasis versus primary tumor (114 out of 265 in G3, 43.0%), suggesting distinct genetic landscapes underpinning various PCa subtypes.

### DiCE genes overlooked by canonical DEA

**Figure 5.**
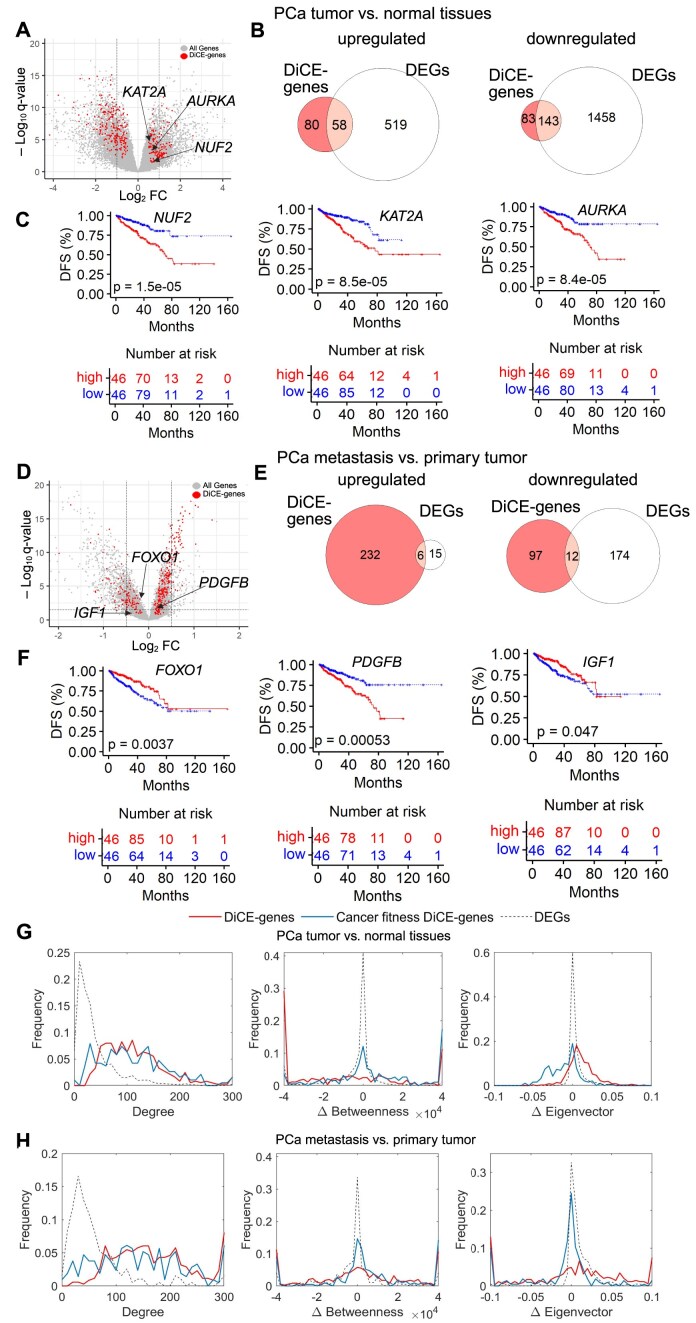
DiCE genes not identified by traditional DEA. (**A**) Volcano plot highlighting three DiCE genes with |log_2_FC| < 1 between PCa tumors and normal tissues. (**B**) Comparisons between DiCE genes and DEGs. (**C**) KM plots of three genes selected in panel (A). (**D**) Volcano plot for PCa metastasis versus primary tumors, showing three genes with subtle gene expression changes. (**E**) Comparisons of DiCE genes and DEGs in the metastasis context. (**F**) KM plots for three DiCE genes highlighted in panel (D). Distribution of degree, Δbetweeness, and Δeigenvector centrality for DiCE genes, cancer fitness DiCE genes, and DEGs, based on analyses of PCa tumor versus normal tissues (**G**) and PCa metastasis versus primary tumors (**H**), respectively.

Traditional DEG analysis typically focuses on genes that exhibit the most significant changes in expression levels. Genes that do not meet certain statistical criteria may be overlooked. However, cancer is a complex disease, and genes involved in its progression may have subtle or context-dependent roles that are not reflected in simple changes in expression levels. For example, 163 out of 364 DiCE genes determined by the comparison between PCa tumor and normal samples cannot pass the typical thresholds for DEGs, FDR < 0.05 and |log_2_FC| > 1 (Fig. 5A), comprising 80 upregulated and 83 downregulated genes (Fig. 5B).

These DiCE genes were enriched in dysregulation of pathways common in PCa and contribute to aggressiveness and treatment resistance, suggesting their important roles in tumor progression and potential as therapeutic targets in PCa. It is important to note that the top-ranked DiCE genes do not necessarily exhibit the highest statistical significance or the largest FCs in expression. Some key genes might have been excluded from the DEG list due to the stringent cutoff criteria typically used in standard analyses, such as *KAT2A*, *NUF2*, and *AURKA* (Aurora kinase A) with relatively modest log_2_FC values of 0.52, 0.69, and 0.70, respectively.

*KAT2A* plays a crucial role in PCa by modulating AR activity through acetylation. Its overexpression correlates with resistance to hormonal therapy, contributing to disease progression and poor clinical outcomes [36]. Targeting *KAT2A* holds a promising strategy to overcome treatment resistance in CRPC [36]. Similarly, dysregulation of *AURKA* plays a crucial role in the tumorigenesis and development of various cancers, including PCa [37]. The observed overexpression of *AURKA* in CRPC samples, especially in cases with amplification of the *AR* gene or elevated *AR* expression levels, highlights its potential significance as a therapeutic target in *AR*-positive CRPC [38, 39]. *NUF2*, a component of the NDC80 kinetochore complex, is essential for proper chromosome segregation during the mitotic process [40]. While its direct involvement in PCa remains underexplored, studies in other cancers suggest its role in genomic instability and tumor progression, including breast cancer, lung cancer, and clear cell renal cell carcinoma [41–43]. Aberrant *NUF2* expressions or activities may contribute to PCa aggressiveness, metastasis, or therapeutic resistance, warranting further investigation.

Notably, 13 of the 47 hub genes previously reported (Fig. 2D) were not classified as DEGs due to their |log_2_FC| being <1 (Supplementary Fig. S3A). Moreover, many of these genes were significantly associated with survival outcomes (Fig. 5C). In fact, 49 out of 134 survival-related DiCE genes exhibited only minimal FCs in expression (|log_2_FC| < 1), a detail that might be missed by conventional DEG methods. Importantly, the identification of these genes by DiCE underscored their potential as PCa biomarkers, highlighting the effectiveness of our approach in uncovering critical genes with subtle yet meaningful contributions to disease progression that may be overlooked by traditional approaches.

The comparison between metastatic and primary PCa tumors also revealed that nearly 95% of DiCE genes were not identified as DEGs (Fig. 5D and E) due to their modest FC magnitudes (|log_2_FC| < 1) or non-significant *q*-values (*q*> 0.05), including 16 out of the 19 hub genes in Fig. 3B (Supplementary Fig. S3B). Particularly, 238 out of 347 DiCE genes exhibited minimal expression shifts (log_2_FC < 0.5) with many falling below 0.2 (Fig. 5D). These included top-ranked genes such as *CDC20* (rank #1, log_2_FC = 0.45; Fig. 4E) and survival-associated genes like *FOXO1* and *PDGFB* (Fig. 5F). We also observed 23 DiCE genes with *q*-values >0.05 in DEA (Fig. 5D), likely due to expression variations or tumor heterogeneity. Among them, *IGF1* was strongly associated with patient survival outcomes (Fig. 5F), consistent with previous reports supporting its important role in PCa [44]. More examples include *CDK13* (*q* = 0.076), *CSNK2A2* (*q* = 0.093), *DECR1* (*q* = 0.072), *PARP1* (*q* = 0.069), and *PRMT5* (*q* = 0.13). Studies have shown that *CDK13* promotes lipid deposition and PCa progression by stimulating NSUN5-mediated m5C modification of *ACC1* mRNA [45].

*CSNK2A2* has been reported as a marker of PCa progression and prognosis [46], while *DECR1* acts as an androgen-repressed survival factor that regulates PUFA oxidation to protect prostate tumor cells from ferroptosis [47]. *PARP1* is a well-established therapeutic target in cancers including PCa, especially in patients harboring BRCA mutations [48]. *PRMT5* overexpression has been closely linked to *AR* expression at both the protein and mRNA levels. Knockdown of *PRMT5* specifically inhibited the growth of PCa cells in an AR-dependent manner and suppressed *AR* transcription [49, 50].

DiCE did not recover certain DEGs identified by conventional DEA (Fig. 5 B and E). For PCa tumor versus normal samples, 519 upregulated DEGs exclusively identified by conventional DEA but missed by DiCE were significantly enriched in the pathways such as Alcoholism and Neuroactive ligand–receptor interaction (Supplementary Fig. S4A). In contrast, 1458 uniquely downregulated DEGs showed enrichment in Metabolic pathways, Cardiac muscle contraction, Chemical carcinogenesis—DNA adducts, and Steroid hormone biosynthesis (Supplementary Fig. S4B). Among them, only Steroid hormone biosynthesis is relevant to PCa, while the rest are generally broad, ubiquitous, or indirectly related. In the PCa metastasis versus primary tumor comparison, 15 uniquely upregulated DEGs were overrepresented in Alcoholism, Systemic lupus erythematosus, African trypanosomiasis, Neutrophil extracellular trap formation, and Shigellosis (Supplementary Fig. S5A), while 174 uniquely downregulated DEGs in metastatic PCa were enriched in the pathways included Cytoskeleton in muscle cells, Dilated cardiomyopathy, Hypertrophic cardiomyopathy, cGMP-PKG signaling pathway, Vascular smooth muscle contraction, Arrhythmogenic right ventricular cardiomyopathy, Regulation of actin cytoskeleton, and Adrenergic signaling in cardiomyocytes (Supplementary Fig. S5B). None of them are inherently specific to PCa biology. Together, these findings emphasize the limitations of relying solely on conventional DEA for identifying functionally important genes.

As previously identified, 36.8% of DiCE genes distinguishing PCa tumors from normal tissues were significantly associated with survival outcomes, a considerably higher proportion than observed for DEGs (23.1%). Among the 134 survival-associated DiCE genes, 76 exhibited a hazard ratio (HR) <1, indicating that lower expression levels were linked to improved survival. Notably, 71 out of these 76 DiCE genes were upregulated in PCa tumors compared to adjacent normal tissues. Conversely, 51 of the 58 DiCE genes with HR >1 were downregulated in PCa tumors. Even more strikingly, 154 out of 155 DiCE genes with HR <1 were upregulated in PCa metastasis compared to primary tumors, while all 22 DiCE genes with HR >1 were downregulated in metastatic tumors.

### Enrichment of cancer fitness genes in DiCE genes

Cancer fitness genes play a critical role in enabling cancer cells to withstand various forms of cellular stress. These genes support cellular endurance in hostile microenvironments, such as during rapid proliferation or the early stages of metastasis, where nutrients, oxygen, and structural support are often limited. While classical oncogenes and tumor suppressor genes primarily drive cell proliferation and genomic instability, cancer fitness genes help sustain cell viability amid physiological challenges. Both categories of genes contribute to cancer progression and lethality. Notably, cancer fitness genes have recently emerged as a distinct and important class in cancer biology. Large-scale functional screens, such as CRISPR (clustered regularly interspaced short palindromic repeats) and RNA interference (RNAi)-based knockout studies, have revealed their roles in tumor survival and adaptation [51–55].

A total of 1927 fitness genes were identified across all three PCa cell lines, 22RV1, DU-145, and LNCaP-Clone-FGC [52]. Among them, 575 and 577 were present in 16 322 and 19 302 expressed genes detected in two datasets used in our study, accounting for 3.5% and 3.0%, respectively. Notably, these PCa fitness genes were significantly overrepresented in the identified DiCE genes, comprising 6.3% (*P* = 2.6 × 10^−3^) and 15.9% (*P* = 1.5 × 10^−24^), respectively, whereas only 0.6% and 1.9% of traditional DEGs were classified as cancer fitness genes. For instance, in the comparison between PCa tumor and normal samples, DiCE identified 61 cancer fitness genes, including *MYC*, *CCNA2*, *PLK1*, *PTTG1*, *EPCAM*, and *MTOR*, that were overlooked by typical DEA but are well-documented contributors to PCa progression (Supplementary Table S1) [39, 56–75]. These genes participate in critical biological pathways and cellular processes such as AR signaling, cell cycle regulation, mitosis, DNA replication, and chromosomal stability, which are frequently dysregulated in advanced or metastatic PCa. These findings indicate the functional significance of the DiCE genes in cancer development and progression, offering valuable insights into potential therapeutic targets for precision medicine and cancer treatment.

As expected, DiCE genes, including those classified as cancer fitness genes, demonstrated greater connectivity as hubs within the PPI network compared to DEGs identified by conventional DEA (Fig. 5G and H), even when their expression changes were modest or minimal. They also displayed more pronounced alterations in the network structure, as reflected by two centrality measures such as betweenness and eigenvector centrality (Fig. 5G and H), whereas only a limited fraction of DEGs exhibited comparable network-level changes.

All these findings highlight the advantage of our proposed DiCE approach in pinpointing key genes for distinguishing different conditions or phenotypes, such as tumor versus normal or metastasis versus primary tumors, while also revealing their clinical significance in patient survival outcomes. Certain DiCE genes that might be overlooked by traditional DEA due to typical cutoffs could still play important roles in cancer progression through diverse mechanisms. Their importance lies in their associations with, or coregulation by, other genes within the PPI network, which DiCE can capture through alterations of centrality measures. Hence, DiCE provides a more comprehensive understanding of disease mechanisms and helps uncover novel therapeutic targets.

## Discussion

In this study, we introduced DiCE, a novel computational framework designed for gene prioritization and biomarker discovery without requiring prior knowledge of disease-causing genes. DiCE leverages multiple analytical techniques, including feature selection, topology analysis of sample-specific weighted PPI networks, centrality measures, and ensemble ranking. As a comprehensive framework, DiCE performs well in analyzing gene expression changes within the context of biological networks. Its versatility enables application across a wide range of diseases and biological processes, allowing researchers to utilize the approach with various datasets and experimental designs to enhance gene prioritization and uncover biologically critical genes, termed DiCE genes, for specific conditions or phenotypes.

When applied to two PCa datasets, DiCE demonstrated its robustness, reliability, and effectiveness in identifying and prioritizing two sets of DiCE genes, 364 genes distinguishing PCa tumor from adjacent normal tissues and 347 genes differentiating PCa metastatic from primary tumors, respectively. A total of 80 DiCE genes were common across both datasets. Among the 347 DiCE genes identified in metastatic PCa, 47 overlapped with genes from a meta-analysis of five studies, indicating their potential role in PCa metastasis, particularly in bone, lymph node, and liver metastasis. Notably, 19 of these DiCE genes were reported as hub genes in the same meta-analysis, yet only 3 of them were determined as DEGs by conventional DEA. This reinforces the effectiveness and reliability of the DiCE approach.

DiCE also successfully captured well-known PCa biomarkers, such as *AR*, *TOP2A*, and *TPX2*, along with a significant number of potential marker genes, like *KAT2A*, *AURKA*, *NUF2*, *CDC20*, *PDGFB*, *IGF1*, and FOXO1. These genes, despite exhibiting smaller FC magnitudes or lower statistical significance, may be missed by conventional thresholds of DEG analysis methods. This demonstrates DiCE’s ability to identify both prominent and subtle but biologically meaningful markers.

Importantly, 36.8% and 51.0% of the two sets of DiCE genes, including those with subtle expression changes, showed strong correlations with DFS outcomes, particularly among 82 shared DiCE genes for both studies (75.6%). These results outperformed those achieved by conventional DEG analysis methods. Additionally, 91.8% and 99.4% of these DFS-linked DiCE genes exhibited a strong concordance between their expression changes and HRs. DiCE genes with HR <1 were predominantly upregulated in PCa tumors compared to adjacent normal tissues or in PCa metastasis compared to primary tumors, whereas genes with HR >1 were downregulated.

Cancer fitness genes, which promote tumor cell survival and proliferation by alleviating cellular stress, are promising targets for cancer therapies. One challenge is that they often show only modest changes in expression levels, which makes them difficult to detect by traditional DEA. As a result, many such genes have historically been underappreciated as potential drivers of disease. Unlike conventional DEA, DiCE addresses this limitation by leveraging perturbations in PPI network centrality, such as betweenness and eigenvector measures, which reflect a gene’s changing influence within the molecular network rather than simply its expression level. We found that 6.3% and 15.9% of DiCE genes were identified as PCa fitness genes across all three PCa cell lines, far exceeding 0.6% and 1.9% observed in traditional DEGs, respectively. Although DiCE genes were identified from bulk RNA-seq data in our study, which does not explicitly account for cellular heterogeneity, the marked enrichment underscores the strength of this network-based approach in capturing functionally important yet subtly expressed genes. This systems-level perspective enables DiCE to prioritize genes with broad regulatory impact, including those whose effects are preserved across heterogeneous tumor populations. While single-cell data could enhance specificity, the current findings indicate that DiCE can extract biologically meaningful signals from bulk data. This underscores its utility in identifying genes with clinical relevance, including potential diagnostic and prognostic biomarkers as well as therapeutic targets. Moreover, the results demonstrate that DiCE offers greater precision in identifying cancer and disease relevant genes compared to traditional DEG analysis, which often yields broader, less functionally focused gene lists.

In conclusion, our results indicate the significance of adopting a comprehensive and multidimensional approach to DEA. Relying solely on traditional DEG analysis risks overlooking important genes and underlying mechanisms. By incorporating differential analysis of PPI networks, DiCE provides novel insights into disease biology, offering an advanced strategy for identifying clinically relevant genes. The approach prioritizes genes based on centrality measure changes, rescuing important marker genes that might be missed by conventional methods. Although this study used PCa as a case example, the DiCE framework is not confined to cancer studies. We have also successfully applied DiCE to datasets from other diseases, such as Alzheimer’s disease (unpublished), where DiCE outperformed traditional DEA by identifying critical genes that exhibited significant changes in network centrality despite minimal differences in gene expression levels. DiCE’s versatility and broad applicability make it a powerful tool for studying diverse diseases and biological contexts. However, its success depends on factors such as accuracy, specificity, and completeness of PPI networks, as well as the quality of gene expression data, which can be influenced by variables like sample purity, sampling bias, and collection conditions. Additionally, challenges related to multimodal data and heterogeneous information may arise. Adopting customized strategies tailored to different data types and integrating them into the analytical pipeline can improve precision and deepen insights into underlying biological mechanisms.

## Supplementary Material

gkaf609_Supplemental_File

## Data Availability

All analyzed data are available from the authors upon request.

## Supplementary data

Supplementary data is available at NAR online.
