# Supplementary material for: DiCE: differential centrality-ensemble analysis based on gene expression profiles and protein–protein interaction network
Source: Nucleic Acids Res. 2025 Jul 8;53(13):gkaf609. doi: 10.1093/nar/gkaf609 (PMC12235518; doi:10.1093/nar/gkaf609)
Supplement: gkaf609_Supplemental_File [file gkaf609_supplemental_file.pdf]

DiCE: differential centrality-ensemble analysis based on gene expression profiles and protein-protein interaction network

Elnaz Pashaei [1], Sheng Liu[1], Kailing Li[2], Yong Zang[3,4,5], Lei Yang[4,6], Tim Lautenschlaeger[5,7], Jun Huang[8], Xin Lu[5,9], Jun Wan[1,2,4,5]

[1]Department of Medical and Molecular Genetics, Indiana University School of Medicine, Indianapolis, IN 46202, USA

[2]Department of BioHealth Informatics, Luddy School of Informatics and Computing, Indiana University at Indianapolis, IN 46202, USA

[3]Department of Biostatistics and Health Data Science, Indiana University School of Medicine, Indianapolis, IN 46202, USA

[4]Center for Computational Biology and Bioinformatics, Indiana University School of Medicine, Indianapolis, IN 46202, USA

[5]Indiana University Simon Comprehensive Cancer Center, Indiana University School of Medicine, Indianapolis, IN 46202, USA

[6]Department of Pediatrics, Herman B Wells Center for Pediatric Research, Indiana University School of Medicine, Indianapolis, IN 46202, USA

[7]Department of Radiation Oncology, Indiana University School of Medicine, Indiana University School of Medicine, Indianapolis, IN 46202, USA

[8]Pritzker School of Molecular Engineering, University of Chicago, Chicago, IL 60637, USA.

[9]Department of Biological Sciences, Boler-Parseghian Center for Rare and Neglected Diseases, Harper Cancer Research Institute, University of Notre Dame, Notre Dame, IN 46556, USA

Correspondence to: Jun Wan, Ph.D., Department of Medical and Molecular Genetics, Indiana University School of Medicine; 410 W. 10th St., HITS 5013, Indianapolis, IN 46202, USA; Phone: (+1) (317) 278-6445; Email: junwan@iu.edu

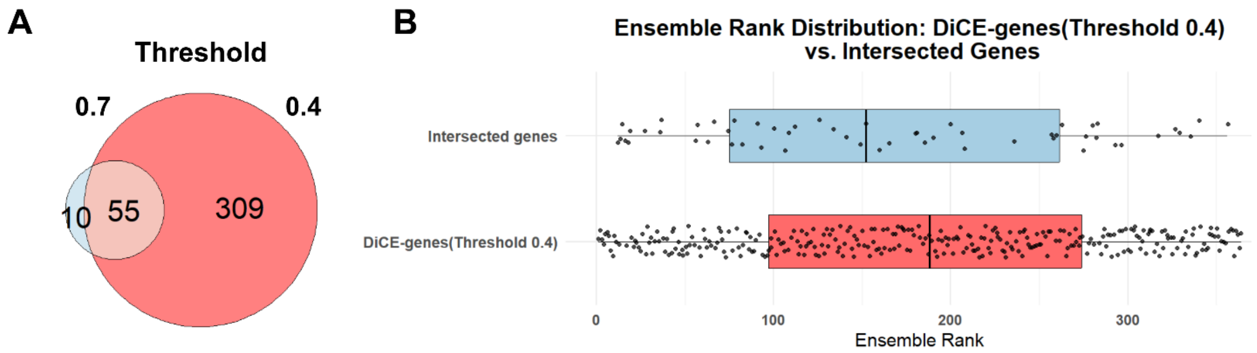

Figure S1. Robust results of DiCE framework under a stringent STRING confidence threshold (0.7 compared to original 0.4 in the main text). (A) Venn diagram showing overlap between DiCE-genes identified using the STRING medium confidence threshold of 0.4 and a stricter threshold of 0.7. Among the 66 genes identified at the higher threshold, 55 were also present in the original set. **(B)** Boxplot comparing ensemble rank distributions of the 55 overlapping genes versus the DiCE-genes only identified based on the 0.4-threshold. Overlapping genes exhibited higher ranks, suggesting stronger interaction support. These results confirm the robustness and reproducibility of DiCE outputs across varying PPI network stringency levels.

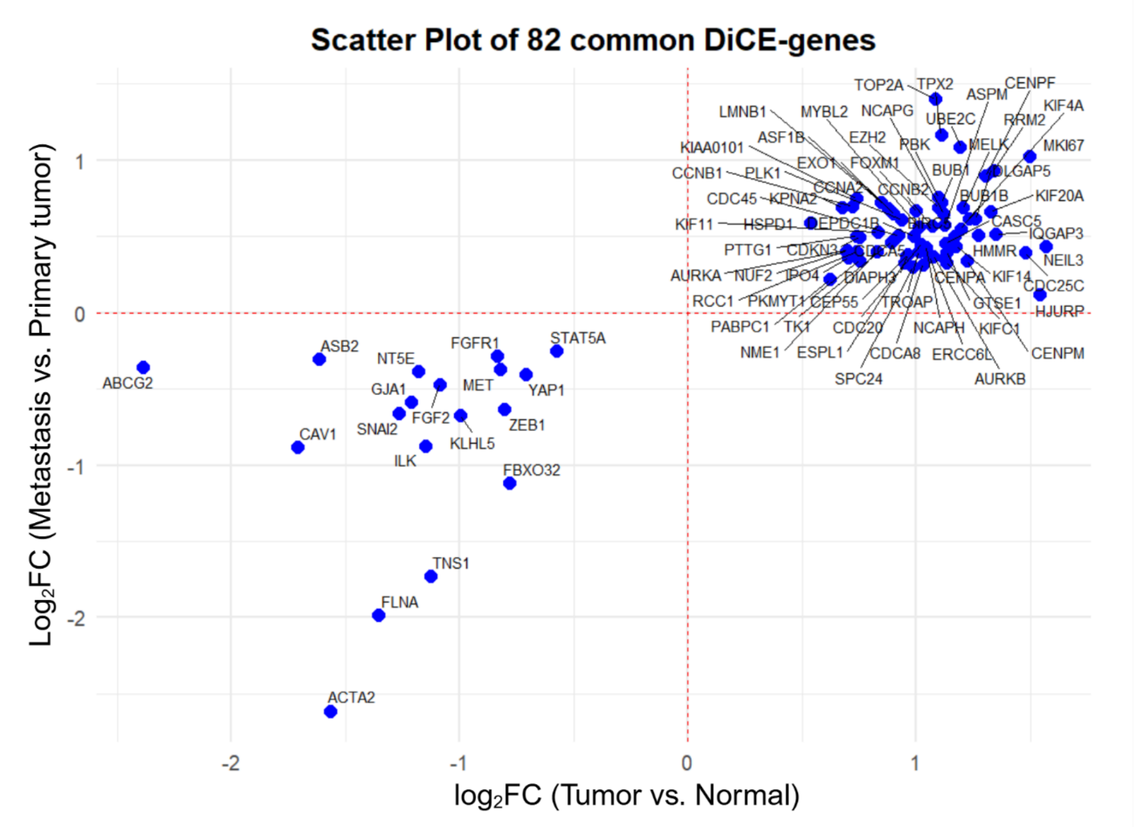

Figure S2. Consistent regulation patterns of DiCE-genes shared between tumor initiation and progression comparisons. Scatter plot shows the log_2FC of 82 DiCE-genes identified in both PCa tumor vs. normal and metastasis vs. primary tumor comparisons. All genes exhibited consistent regulation directions, either upregulated or downregulated in both contrasts, suggesting their potential roles across both tumor initiation and progression.

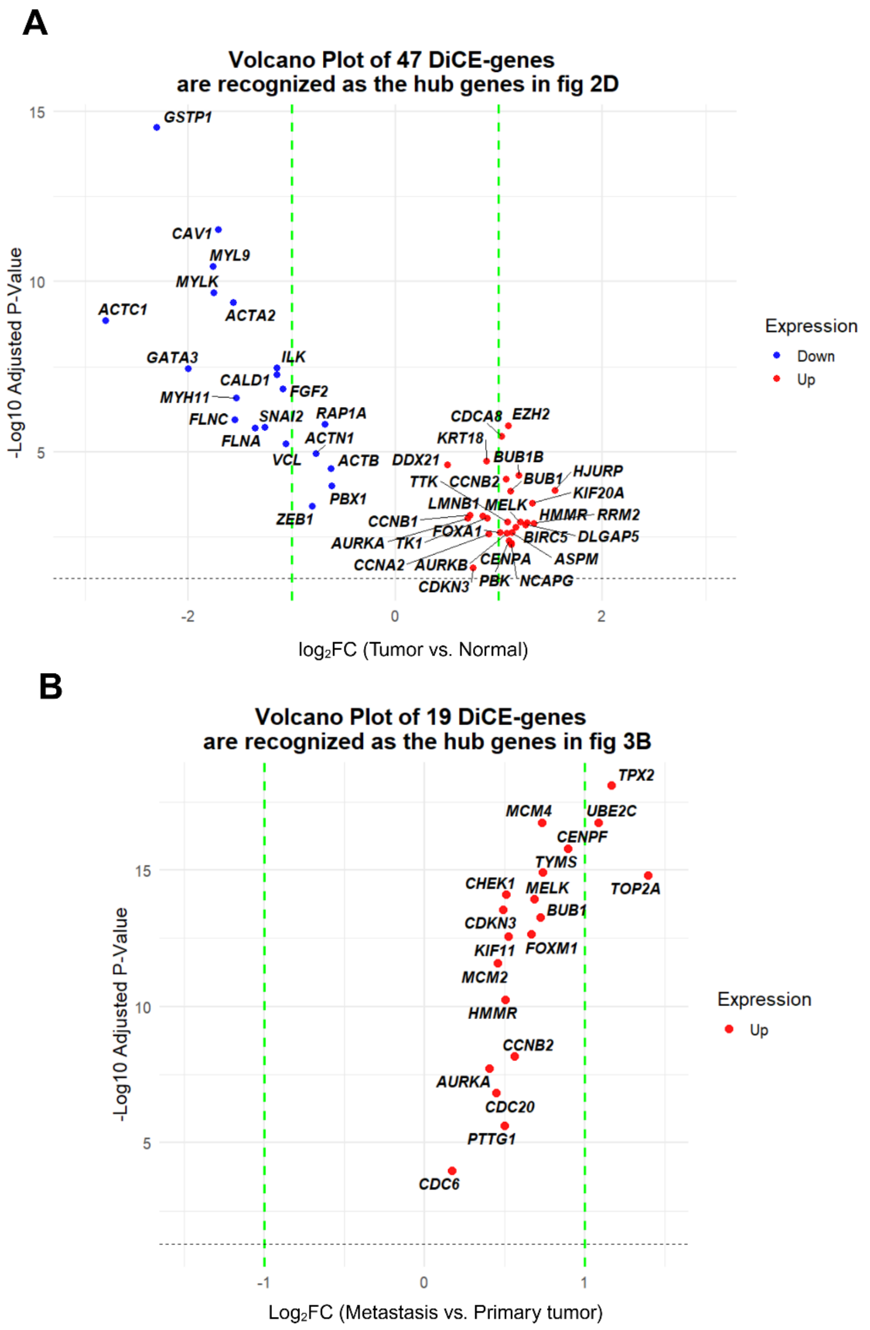

Figure S3. Volcano plot of DiCE-genes confirmed as hub genes in PCa by previous studies. **(A)** Log_2FC values of 47 DiCE-genes identified as hub genes in Fig. 2D. Of these, 13 did not meet the conventional DEA threshold of $|log_2FC| > 1$. **(B)** Log_2FC values of 19 DiCE-genes reported as hub genes in Fig. 3B, with only 3 classified as DEGs ($|log_2FC| > 1$). These results highlight DiCE's ability to capture both previously recognized and novel hub genes that may be missed by traditional DEG-based approaches.

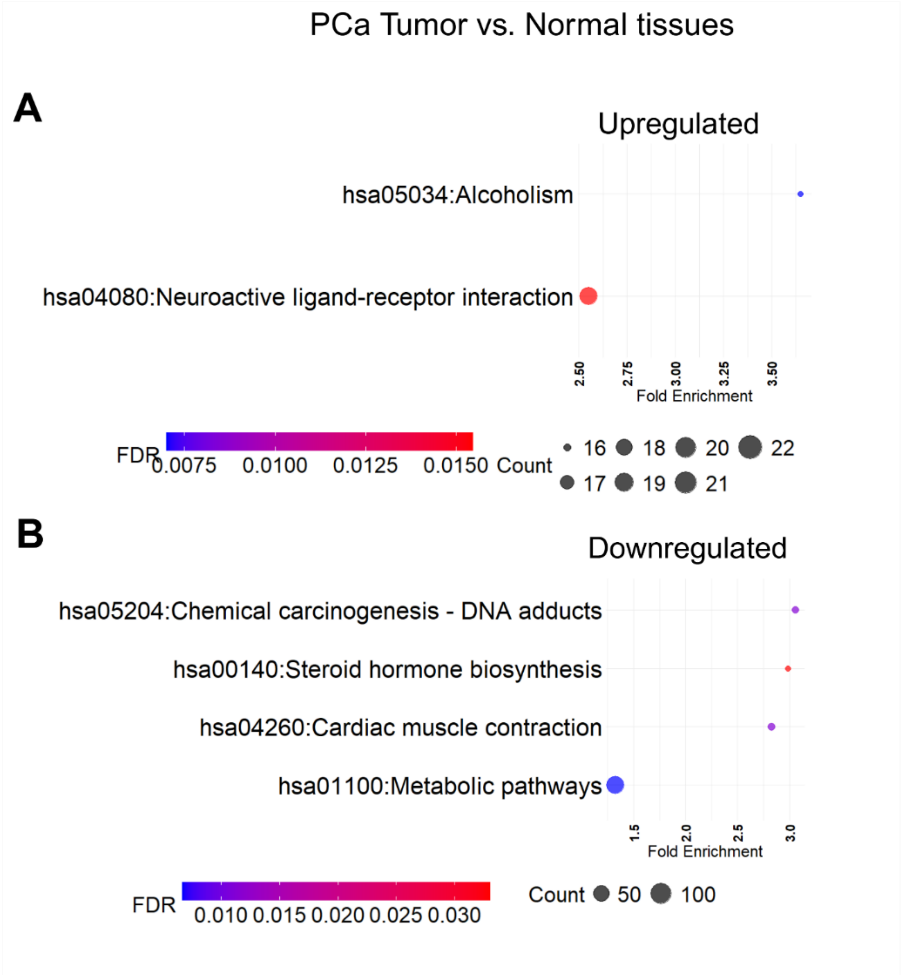

Figure S4. Pathways enriched in DEGs exclusively identified by conventional DEA and classified as (A) up-regulated or (B) down-regulated in PCa tumors relative to normal samples.

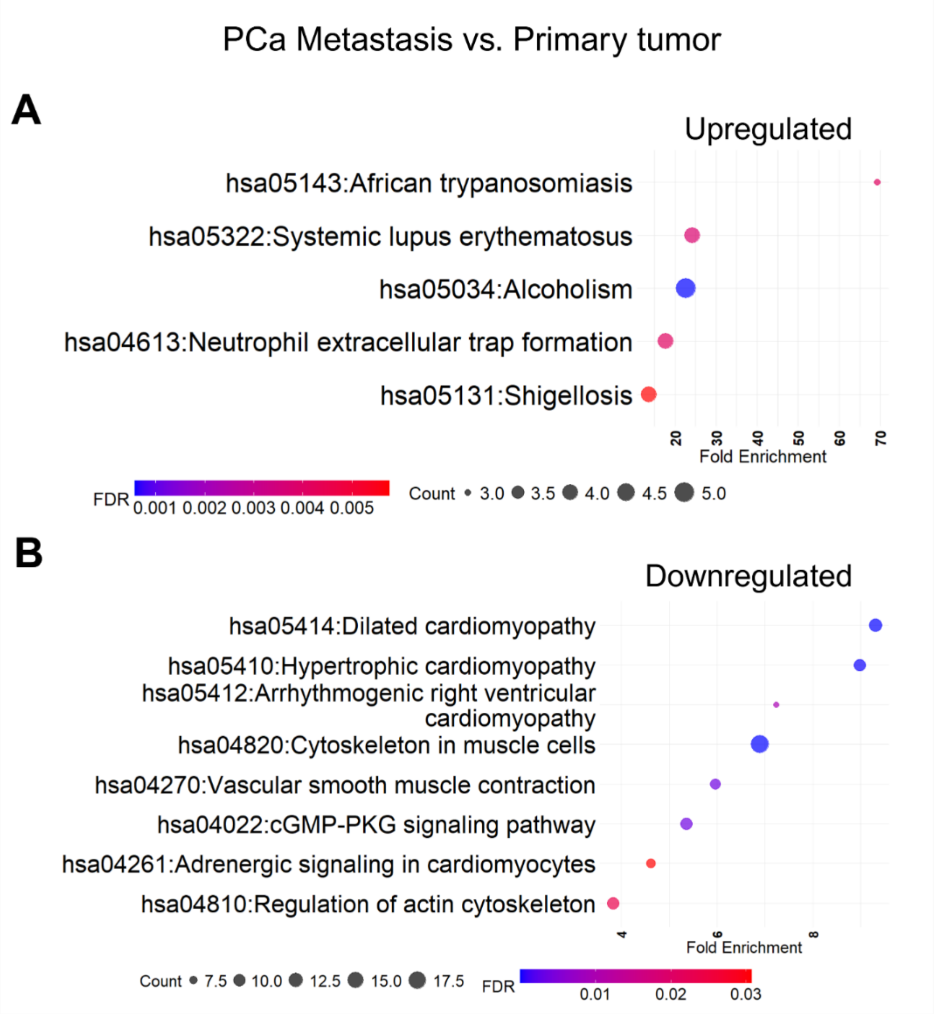

Figure S5. Pathways enriched in DEGs exclusively identified by conventional DEA and classified as (A) up-regulated or (B) down-regulated in PCa metastasis vs. primary tumor.

Table S1. Selected cancer fitness genes prioritized by DiCE with functional relevance to PCa progression. Genes marked in red were not detected as DEGs in this study.

Official Gene Symbol	Function	PCa Relevance	Reference
ANAPC4	Subunit of the anaphase-promoting complex; controls metaphase–anaphase transition.	Dysregulation linked to chromosomal instability in PCa.	[56]
AURKA	Kinase regulating mitotic progression.	Driver of PCa progression; promising targeted therapy.	[57]
AURKB	Mitotic kinase is essential for cytokinesis.	Overexpression in early PCa; therapeutic target.	[58]
BIRC5	Inhibitor of apoptosis protein promoting cell survival.	Prognostic biomarker in PCa.	[59]
BUB1B	Mitotic checkpoint protein ensures proper chromosome alignment.	Overexpression predicting poor PCa outcomes.	[60]
CASC5 (KNL1)	Chromosome segregation protein.	High expression linked to poor prognosis and immune infiltration.	[61]
CCNA2	Regulator of G1/S and G2/M cell cycle transitions	Promoting PCa progression; association with higher Gleason score, recurrence, and poor prognosis.	[62]
CCNB1	Essential regulator of G2/M cell cycle transition	Overexpression linked to PCa aggressiveness, therapy resistance, and poor prognosis	[39]
CDC20	Activator of APC/C complex during mitosis.	Driver of PCa progression via β-catenin stabilization.	[63]
CDC45	Component of CMG helicase; initiates DNA replication.	Biomarker for metastasis and therapeutic target in PCa.	[64]
DDX21	RNA helicase with transcriptional, rRNA processing, and RNA structural modulation roles.	Abnormal expression affecting PCa cell proliferation, invasion, and apoptosis.	[65]
EPCAM	Cell adhesion molecules involved epithelial cell signaling.	Overexpression in both localized and metastatic PCa; association with epithelial-mesenchymal transition and metastasis.	[66],[67]
GNL3	GTP-binding protein involved in ribosome biogenesis and cell proliferation.	Overexpression correlating with PCa progression and poor survival outcomes; potential prognostic biomarker.	[68]
KIF20A	Kinesin-like protein is involved in mitosis and vesicle transport.	Progression promotion to castration-resistant PCa via AR activation.	[69]
MTOR	Serine/threonine kinase regulating cell growth and metabolism.	Activation of the PI3K/Akt/mTOR pathway contributes to PCa progression; therapeutic potential of mTOR inhibitors	[70]
MYC	Transcription factor regulating cell proliferation and growth.	Overexpression disrupting androgen receptor signaling, promoting PCa initiation and progression.	[71]
PLK1	Serine/threonine kinase involved in cell cycle progression.	Overexpression linked to PCa progression; potential therapeutic target.	[72]
PTTG1	Securin protein regulating sister chromatid separation	Elevated expressions associated with PCa progression and metastasis.	[73]
TLN1	Cytoskeletal protein involved in integrin activation.	Overexpression of TLN1 promoting cell adhesion, migration, and invasion in PCa cells.	[74]
TOP2A	DNA topoisomerase is essential for replication and transcription.	Overexpressed in aggressive PCa; valuable prognostic/therapeutic marker.	[75]
